# Diagnostic Performance of Dual-Energy Subtraction Radiography for the Detection of Pulmonary Emphysema: An Intra-Individual Comparison

**DOI:** 10.3390/diagnostics11101849

**Published:** 2021-10-07

**Authors:** Julia A. Mueller, Katharina Martini, Matthias Eberhard, Mathias A. Mueller, Alessandra A. De Silvestro, Philipp Breiding, Thomas Frauenfelder

**Affiliations:** 1 Institute of Diagnostic and Interventional Radiology, University Hospital Zurich, 8091 Zürich, Switzerland; juliaanna@gmx.ch (J.A.M.); Matthias.eberhard@gmx.de (M.E.); desilvestro.alessandra@usz.ch (A.A.D.S.); philipp.breiding@usz.ch (P.B.); Thomas.frauenfelder@usz.ch (T.F.); 2 Institute of Radiology, Cantonal Hospital of Frauenfeld, 8501 Frauenfeld, Switzerland; Mahias.mueller@stgag.de

**Keywords:** lung, conventional radiography, diagnostic procedure, chronic obstructive pulmonary disease

## Abstract

Purpose/Objectives: To compare the diagnostic performance of dual-energy subtraction (DE) and conventional radiography (CR) for detecting pulmonary emphysema using computed tomography (CT) as a reference standard. Methods and Materials: Sixty-six patients (24 female, median age 73) were retrospectively included after obtaining lateral and posteroanterior chest X-rays with a dual-shot DE technique and chest CT within ±3 months. Two experienced radiologists first evaluated the standard CR images and, second, the bone-/soft tissue weighted DE images for the presence (yes/no), degree (1–4), and quadrant-based distribution of emphysema. CT was used as a reference standard. Inter-reader agreement was calculated. Sensitivity and specificity for the correct detection and localization of emphysema was calculated. Further degree of emphysema on CR and DE was correlated with results from CT. A *p*-value < 0.05 was considered as statistically significant. Results: The mean interreader agreement was substantial for CR and moderate for DE (k_CR_ = 0.611 vs. k_DE_ = 0.433; respectively). Sensitivity, as well as specificity for the detection of emphysema, was comparable between CR and DE (sensitivity_CR_ 96% and specificity_CR_ 75% vs. sensitivity_DE_ 91% and specificity_DE_ 83%; *p* = 0.157). Similarly, there was no significant difference in the sensitivity or specificity for emphysema localization between CR and DE (sensitivity_CR_ 50% and specificity_CR_ 100% vs. sensitivity_DE_ 57% and specificity_DE_ 100%; *p* = 0.157). There was a slightly better correlation with CT of emphysema grading in DE compared to CR (r_DE_ = 0.75 vs. r_CR_ = 0.68; *p* = 0.108); these differences were not statistically significant, however. Conclusion: Diagnostic accuracy for the detection, quantification, and localization of emphysema between CR and DE is comparable. Interreader agreement, however, is better with CR compared to DE

## 1. Introduction

Chronic obstructive pulmonary disease (COPD) is defined symptomatically as chronic bronchitis and physiologically as airway obstruction or anatomically as emphysema [1], usually caused by tobacco use [2]. Its course is creeping and progressive with a high impairment in quality of life [3] and COPD is a leading cause of death worldwide [4]. The early detection of emphysematous lung tissue is important to prevent and manage the global disease burden [5,6].

Since the pathogenesis in COPD is not fully understood and pulmonary function tests (PFT) are not sensitive in detecting mild emphysema and fail to register the heterogeneity of the disease, radiological imaging plays a major role in emphysema detection and evaluation [7,8]. Computed tomography (CT) is the most sensitive radiological imaging modality for the detection, quantification, and phenotyping of emphysema [9,10]. Due to the high sensitivity of HRCT, pulmonary emphysematous changes detected before PFT (forced expiratory volume in 1 s, FEV1) are pathologic [11]. The benefit of earlier therapy of chronic cough with normal FEV1 but conspicuous features in CT is not yet known, but a delay of disease progression is postulated [12]. Conventional radiography (CR) seems not to be as reliable as CT in the detection of emphysema unless the disease is advanced. Indirect signs, such as horizontal standing ribs, extended intercostal space, flattened diaphragms, retrosternal air space, increased radiographic transparency, and rarefication of small blood vessels in the periphery, can give a hint to the underlying disease, however [13].

Nevertheless, CR holds its position as a first diagnostic approach in the daily clinical practice due to its broad availability; fast examination time; low cost and low radiation dose [14,15]; and new developments, such as dual energy subtraction chest X-ray (DE), which might help to increase its diagnostic accuracy.

In DE, besides the standard image, a soft tissue image with bone information removed and a bone image with soft tissue information removed is generated [16,17].

Previous studies have shown that DE images improve the sensitivity for shading lesions, such as the detection of infectious consolidations, tumors, interstitial lung changes, and aortic or tracheal calcification, compared to CR-images [18,19,20,21]. We hypothesize that DE might improve the conspicuousness of hyperlucent lung pathologies in a similar way.

Therefore, the aim of this study was to assess the diagnostic performance of DE for detecting pulmonary emphysema compared to CR using CT as a reference standard.

## 2. Materials and Methods

### 2.1. Patient Population

The study was approved by the institutional review board and local ethics committee (KEK Zürich: Cantonal ethics committee Zurich Switzerland). Informed consent was waived because of the retrospective setting of this study (blinded for review).

In this observational study, 74 patients (age: 71.6 ± 8.7 years, 26 females) undergoing CR, DE, and chest CT between September 2015 and Mai 2019 were retrospectively included. Inclusion criterion was the presence of a CT ± 3 months within the conventional imaging. Patients were excluded when there was an intervention (interventional or surgical lung resection) between imaging. In- and exclusion criteria are listed in Table 1.

### 2.2. Data Acquisition

#### 2.2.1. CR and DE Images

**Figure 1:**
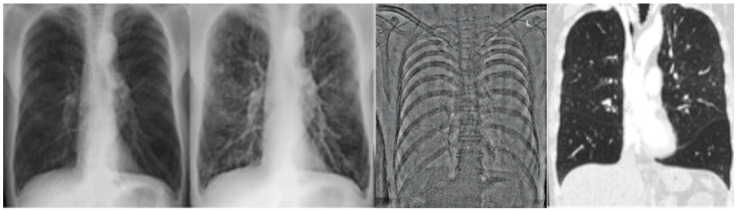
Moderate emphysema, right upper lobe and left lower lobe. Left: conventional x-ray postero anterior (p.a.), middle: dual energy x-ray p.a., right: corresponding computed tomography image.

All patients underwent chest radiography in lateral and p.a. projection, whereby the latter was obtained using a dual energy mode (FDR AcSelerate, Fujifilm, Tokyo, Japan) at a tube current of 7 mA and a tube voltage of 120 kV and 60 kV after a delay of 150 ms according to institution’s standard protocol. The higher energy exposure was used to produce the CR image. With the use of a post-processing algorithm, the “virtual” soft tissue and bone image were calculated from the two acquisitions (see Figure 1).

#### 2.2.2. CT Images

Single-energy CT was performed with or without intravenously injected contrast agent at 120 kV/110 mA (Somatom Sensation, Somatom Flash and Somatom Force, Siemens Healthcare, Forchheim, Germany) and reconstructed with a slice thickness of 2.0 mm. A radiation dose was recorded for each scan.

### 2.3. Image Analysis

#### 2.3.1. CR/DE Image Analysis

All images were anonymized prior to readout. Two experienced readers (5 and 10 years of experience in thoracic imaging, respectively) reviewed the images in two reading-rounds:

Reading-round 1 (CR): Only the conventional p.a. and lateral projections were evaluated.

Reading round 2 (DE): All images (including the p.a. bone and soft tissue images) of the patients were evaluated.

**Figure 2:**
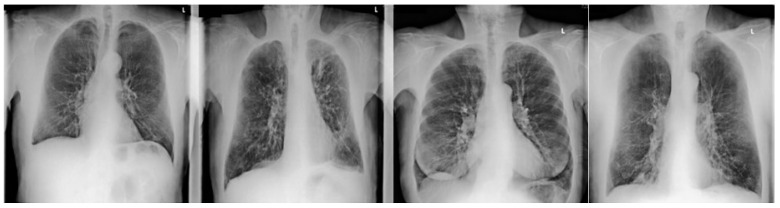
Dual energy soft tissue x-ray image of non-mild-moderate-severe emphysema in the right upper lobe.

**Figure 3:**
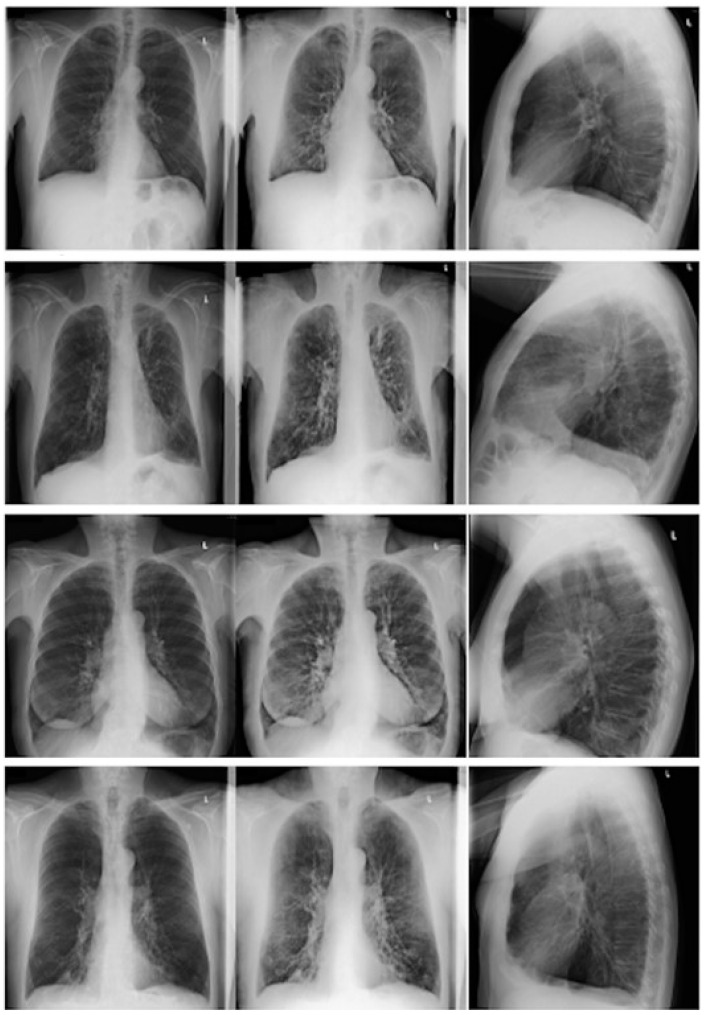
Four patients with varying degrees of emphysema in the right upper lobe.First row: no emphysema; second row: mild emphysema; third row: moderate emphysema; and fourth row: severe emphysema. Left column: conventional postero anterior (p.a.) x-ray; middle column: dual-energy p.a. x-ray; and right column: conventional lateral x-ray.

Readers had to evaluate the images for the presence (yes/no) of emphysema. If emphysema was present, readers had to score the degree of emphysema (none, mild, moderate and strong; 1–4) of emphysema. For the quantification of emphysema, readers were trained with four data sets showing the entire range of emphysema manifestations (Figure 2 and Figure 3).

Readers further had to sign the quadrant (upper right, lower right, upper left, lower left) of the most affected area.

If there was a disagreement between the two readers, re-evaluation was performed until consensus was sought. The time frame between the two reading-rounds was of four weeks to avoid a recall bias.

#### 2.3.2. CT Image Analysis

**Figure 4:**
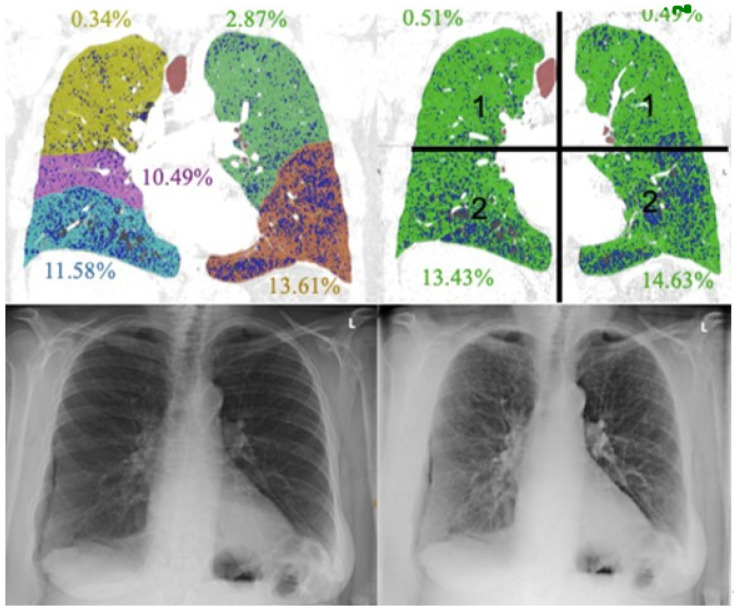
Lobe- and quadrant-based quantification.

One expert reader (15 years of experience in thoracic imaging) evaluated the CTs for the presence (yes/no) of emphysema. Further evaluation of CT images was performed with a commercially available software tool (Ziostation, Ziosoft Inc., Tokyo, Japan). The software quantified emphysema using the Goddard score (Figure 4) and assessed the ratio of low attenuation area to lung volume (LAA%) using a threshold of −950 Hounsfield units (HU).

The Goddard score is a semi-quantitative assessment score functioning as a surrogate marker for the presence of emphysema based on the evaluation of low attenuation areas in a number of representative lung fields. The total score is defined as the sum of the single scores [22]. The Goddard score was used for overall emphysema grading as well as for defining the most affected lung quadrant.

#### 2.3.3. Statistical Analysis

Statistical analysis was conducted using SPSS (released 2017, version 25.0, Armonk, NY, USA). Interreader agreement for binomial variables was calculated with Cohen’s kappa (*κ*). According to Landis and Koch [23], *κ* values were defined as follows: slight agreement (*κ* = 0–0.2), fair agreement (*κ* = 0.21–0.40), moderate agreement (*κ* = 0.41–0.60), substantial agreement (*κ* = 0.61–0.80), and almost perfect agreement (*κ* = 0.81–1.0). For calculating the interreader agreement in emphysema grading, the interclass correlation coefficient (ICC) was used. An ICC below 0.5 was considered as poor agreement, values between 0.5 and 0.75 were considered as moderate, and values between 0.75 and 0.9 were considered as good agreement [24].

Sensitivity, specificity, negative predictive value (NPV), and positive predictive value (PPV) for the presence and location of the most affected quadrant were calculated. Further, emphysema grading on CR and DE were correlated with the CT-derived Goddard score using Pearson’s correlation (*r*). A *p*-value of <0.05 in overall analysis was considered significant.

## 3. Results

### 3.1. Patient Population

Indications for conventional radiography and chest CT in the included patents were medical evaluation of potential lung volume reduction surgery (LVRS; *n* = 23), further assessment of radiological findings (e.g., pulmonary noduli or malignancies) or clarification of clinically persistent symptoms (e.g., chronical cough, recurrent infections or hemoptysis; *n* = 32), and vascular indications (*n* = 10).

A total of eight participants were excluded due to the following events within the time interval between CT and DE: lung volume reduction surgery (*n* = 1), new small pleural effusion (*n* = 1), new pneumothorax (*n* = 1), progressive lymphatic spread of leukemia (*n* = 1), evolution of pericardial effusion (*n* = 1), paraseptal emphysema (*n* = 2), and bilateral discrete emphysema (*n* = 1).

### 3.2. CT Images: Standard of Reference

The time interval between CT and conventional radiography was of 28 ± 58 days (see Table 2).

### 3.3. Presence of Emphysema

From the 66 included patients, 81.8% (*n* = 54) showed emphysema.

### 3.4. Emphysema Grading

The mean Goddard score based on quantitative CT analysis was 7 (SD ± 4.5; range 0–22). The mean LAA% based on quantitative CT analysis was 16.66% (SD ± 17.1; range 0.01–82.3%). Thirty-two patients had mild emphysema (Goddard-Score 1–7), twenty had moderate emphysema (Goddard-Score 8–15), and two had severe emphysema (Goddard-Score >15).

### 3.5. CR and DE Image Analysis

#### 3.5.1. Interreader Agreement

Overall, the mean interreader agreement was substantial for CR and moderate for DE (k_CR_ = 0.611 vs. k_DE_ = 0.433; respectively). While the interreader agreement was comparable for emphysema grading (both good), the interreader agreement for the presence of emphysema and for the assignation of the most affected lung quadrant was better in CR (substantial and fair) compared to DE (moderate and slight) (see Table 3).

#### 3.5.2. Presence of Emphysema and Location of the Most Affected Lung Quadrant

Sensitivity as well as specificity for the detection of emphysema was comparable between CR and DE (sensitivity_CR_ 96% and specificity_CR_ 75% vs. sensitivity_DE_ 91% and specificity_DE_ 83%; *p* = 0.157). Similarly, there was no significant difference in the sensitivity or specificity for emphysema localization between CR and DE (sensitivity_CR_ 50% and specificity_CR_ 100% vs. sensitivity_DE_ 57% and specificity_DE_ 100%; *p* = 0.157) (Table 4).

#### 3.5.3. Severity of Emphysema between CR/DE and CT

The average subjective emphysema score was rated significantly higher in DE (mean: 2.62 ± 0.87) versus CR (mean: 2.45 ± 0.89; *p* = 0.003; controls included). Emphysema grading with DE showed a slightly higher correlation with the Goddard score than with CR; these differences, however, were not statistically significant (r_DE_ = 0.75 vs. r_CR_ = 0.68; *p* = 0.108). Similarly, emphysema grading with DE showed a slightly higher correlation with LAA% than with CR lacking statistical significance (r_DE_ = 0.73 vs. r_CR_ = 0.71; *p* = 0.586).

## 4. Discussion

We compared DE to CR for the evaluation of lung emphysema, and found that diagnostic accuracy for the detection, quantification, and localization of emphysema between CR and DE is comparable. The interreader agreement, however, was better with CR compared to DE.

Clinically, PFT is used to diagnose COPD. PFT, however, is relatively insensitive to the severity and distribution of emphysema. (1) There is no correlation between reduced FEV1 and severity of lung emphysema, leading to a wide range in severity of emphysema despite having clinically the same disease stage [25]. (2) Clinical presentation of emphysema does not definitively relate to the distribution of emphysema on imaging [26,27,28,29], and upper lung zones are rather silent regions in PFT, leading to a high percentage of patients with mild to moderate disease being missed by PFT [30,31]. (3) FEV1 depends on the patient’s cooperation. These points stress the importance of imaging in early stages of COPD. Further, some patients undergo chest X-ray for other clinical questions (i.e., pre-operative evaluation, evaluation of infective consolidation.) without the suspicion of emphysema or signs of COPD. These patients would otherwise not undergo PFT and could be lost.

Conventional imaging, which is often used as baseline imaging, only yields a moderate sensitivity for detecting emphysema (approximately 40%) [32]. This is due to the slight difference in X-ray absorption of pulmonary parenchyma, resulting in low conspicuity of the disease on conventional imaging [33]. DE is a new imaging modality with the potential to overcome these difficulties. In DE, a post-processing algorithm separates calcium-containing structures from soft-tissue components and overcomes the problem of superimposition of several structures [34].

Further, the less penetrating beam with the lower tube voltage used in DE results in a higher dynamic range of resultant image data, higher intrinsic contrast (i.e., lesion’s intensity relative to the surrounding tissue intensity), and hence a better depiction of the lung parenchyma and its pathology [35].

In fact, previous studies could show that DE improves the sensitivity for shading lesions, such as the detection of infectious consolidations, tumors, interstitial lung changes, and aortic or tracheal calcification compared to CR images [18,19,20,21]. Other studies have shown that DE can reduce diagnostic errors of chest pathologies and prevent misdiagnosis of consolidations or lung nodules, for example, also by less-experienced radiologists [34,36]. The higher accuracy for detecting focal opacities (i.e., lung nodules or infectious infiltrates) was attributed to the better accentuation of lung abnormalities [37]. Since the better intrinsic contrast should yield also higher diagnostic accuracy for hyperlucencent lung pathologies, also called “minus pathologies”, we hypothesized that DE-images emphasize emphysematous lung sections in a similar way and, thus, may aid in earlier detection of pulmonary emphysema.

Our results, however, could not show a higher diagnostic accuracy for the detection and localization of emphysema. On the contrary, interreader agreement seemed to be worse with DE, even though the readers had also the standard CR images side by side when evaluating the DE images. We believe that, quite unusual, soft tissue and bone images confused the readers more in their diagnosis than they helped. Therefore, readers might benefit from training in order to get used to the DE images. Further, differences in the depiction of emphysema might be so subtle that there is no measurable clinical benefit in using DE instead of CR. The results are further hampered by the radiologist inexperience to evaluate DE images, reflected in the worse interreader experience compared to CR.

An interesting observation we made in this study concerns the relatively high sensitivity in the detection of emphysema compared to values reported in the literature for CR [32]. This might be due to the lower kV used for the acquisition of DE images compared to conventional CR images. The higher soft tissue contrast with the lower kV used in DE might yield a better distinction of emphysematous lung changes from normal lung parenchyma. Since CR and the DE images were acquired in our study with the lower kV, both CR and DE benefit from the lower kV and had higher sensitivity for emphysema detection. The acquisition of two consecutive X-Rays, first with a conventional CR and then with the DE technique, in order to compare the sensitivities between a conventional CR and DE, would have been unethical. However, previous studies have shown that the use of lower tube voltages resulting in lower beam penetration enhances density differences in the lung [38].

Subjective emphysema grading for both CR and DE correlated well with CT. Even though we could observe a slightly better correlation of DE with CT than with CR, differences were not statistically significant.

The downsides of DE are definitely the higher radiation dose, which is only partially compensated on lateral chest radiography and the risk of motion artifacts which can occur when the patient moves between the two image acquisitions [16].

Even if CR holds its position in initial chest evaluation, it insufficiently quantifies regional lung perfusion and emphysema, evaluates fissural integrity, or stimulates the effect of surgical resection. Therefore, to guide therapeutic options in extended emphysema (e.g., lung volume reduction surgery, endobronchial valves, coils), further imaging examinations are essential [39].

Dual-energy CT imaging methods can not only gain anatomical information but also functional information too. For example, lung iodine perfusion blood volume (iPBV) illustrates regional lung perfusion changes [40], or inhalative xenon tracer gas functions as a surrogate for regional lung ventilation. These modalities correlate with the degree of emphysema and could serve as tools for detecting mild emphysema [41]. Nevertheless, lowering the radiation dose by displaying only a target volume would render overall assessment impossible [42].

Limitations of the study are as follows. First, the quantification of emphysema was based on a subjective scoring system, which may limit interreader comparability. Second, the degree of emphysema in CR and DE may be underestimated due to soft tissue overlay, especially in corpulent patients. Third, we did not distinguish between different types of emphysema (centrilobular, panlobular, paraseptal). Forth, uneven ventilation or hyperinflation might affect the detection of emphysema. While reduced ventilation could lead to underestimation of emphysema due to denser lung parenchyma, overinflation on the other hand could potentially lead to an overestimation of emphysema. These factors similarly affect CT densitometry based on HU values. Fifth, we did not perform a correlation of our findings with PFT. Due to the retrospective nature of our study, PFT was not available in the majority of patients. Sixth, in our study, both the CR and the DE images were acquired with the lower kV, meaning that we could not show a difference in sensitivity for emphysema detection related to different kV in our dataset. The acquisition of two consecutive X-rays, first with a conventional CR and then with the DE technique, would have been unethical. However, previous studies have shown that the use of lower tube voltages resulting in lower beam penetration enhances density differences in the lung [38].

## 5. Conclusions

In conclusion, diagnostic accuracy for the detection, quantification, and localization of emphysema between CR and DE is comparable. This implies that the presumably higher tissue contrast in DE did not have the expected benefit in the evaluation of emphysema. Besides that, interreader agreement was influenced negatively by the evaluation of DE, which we attribute to the unfamiliarity of the readers with the new technique.

An interesting observation we made in this study was the relatively high sensitivity in the detection of emphysema compared to values reported in the literature for CR [32]. This might be due to the lower kV used for the acquisition of DE images compared to conventional CR images, potentially resulting in a better distinction of emphysematous lung changes from normal lung parenchyma.

## Figures and Tables

**Table 1:** Inclusion and exclusion criteria.

Inclusion Criteria	Exclusion Criteria
Age > 18 years	Incapability of undergoing upright chest radiography
Existence of chest examinations with DE and CT	Cardiopulmonary decompensation
Description of lung emphysema in radiologic findings or COPD in list of diagnosis	Obscured lung tissue by foreign bodies, e.g., cardiac devices
Short time interval between the two imaging modalities	Consolidation, e.g., empyema, encapsulated pneumonia
	Changes between performed DE and reference CTOccurrence of a pneumothorax Lung volume reduction surgery Endoscopic lung volume reduction, e.g., valves, coils, sealants

DE = dual energy subtraction radiography; CT = computed tomography.

**Table 2:** Patient characteristics.

	Patients	Controls
Number	61	13
Age (years), mean ± SD	71.9 ± 8.2	70.6 ± 10.8
Time between CT and CR/DE (days), mean ± SD	41.8 ± 1.4	20.7 ± 46.8
Male:Female ratio	40:21	8:5

SD = standard deviation; CT = computed tomography; CR = conventional radiography; DE = dual energy subtraction radiography.

**Table 3:** Interreader comparison of assessed features.

Assessment Features	Kappa bzw. ICC CR	Kappa bzw. ICC DE
Presence of emphysema (yes/no)	0.693 (substantial)	0.462 (moderate)
Subjective emphysema score (none = 1, mild = 2, moderate = 3, severe = 4)	0.834 (good)	0.809 (good)
Location of maximal emphysema manifestation	0.306 (fair)	0.027 (slight)

ICC = intra-class correlation; CR = conventional radiography, DE = dual energy subtraction radiography.

**Table 4:** Test characteristics of CR and DE.

	Presence of Emphysema		Location of Maximal Emphysema Manifestation	
Assessment parameter	CR	DE	CR	DE
Sensitivity	96.3%	90.7%	50%	57.4%
Specifity	75%	83.33%	100%	100%
NPV	81.82%	66.67%	30.77%	34.29%
PPV	94.55%	96.08%	100%	100%

CR = conventional radiography, DE = dual energy subtraction radiography, NPV = negative predictive value, PPV = positive predictive value.

## Data Availability

The study data is not available in a public database. However, data can be requested at the corresponding author.
